# Meaningful health outcomes for paediatric neurodisability: Stakeholder prioritisation and appropriateness of patient reported outcome measures

**DOI:** 10.1186/s12955-015-0284-7

**Published:** 2015-06-25

**Authors:** Christopher Morris, Astrid Janssens, Valerie Shilling, Amanda Allard, Andrew Fellowes, Richard Tomlinson, Jane Williams, Jo Thompson Coon, Morwenna Rogers, Bryony Beresford, Colin Green, Crispin Jenkinson, Alan Tennant, Stuart Logan

**Affiliations:** PenCRU, Child Health Group, University of Exeter Medical School, University of Exeter, South Cloisters, St Luke’s Campus, Exeter, EX1 2LU UK; PenCLAHRC, University of Exeter Medical School, University of Exeter, South Cloisters, St Luke’s Campus, Exeter, EX1 2LU UK; Council for Disabled Children, National Children’s Bureau, London, UK; Department of Child Health, Royal Devon and Exeter NHS Foundation Trust, Exeter, UK; Department of Child Health and Paediatrics, Nottingham University Hospitals NHS Trust, Nottingham, UK; Social Policy Research Unit, University of York, York, UK; Nuffield Department of Population Health, University of, Oxford, UK; Department of Rehabilitation Medicine, University of Leeds, Leeds, UK

**Keywords:** Children, Neurodisability, Patient reported outcome, Prioritisation, Core outcome set

## Abstract

**Background:**

Health services are increasingly focused on measuring and monitoring outcomes, particularly those that reflect patients’ priorities. To be meaningful, outcomes measured should be valued by patients and carers, be consistent with what health professionals seek to achieve, and be robust in terms of measurement properties.

The aim of this study was (i) to seek a shared vision between families and clinicians regarding key aspects of health as outcomes, beyond mortality and morbidity, for children with neurodisability, and (ii) to appraise which multidimensional patient reported outcome measures (PROMs) could be used to assess salient health domains.

**Methods:**

Relevant outcomes were identified from (i) qualitative research with children and young people with neurodisability and parent carers, (ii) Delphi survey with health professionals, and (iii) systematic review of PROMs. The International Classification of Functioning Disability and Health provided a common language to code aspects of health. A subset of stakeholders participated in a prioritisation meeting incorporating a Q-sorting task to discuss and rank aspects of health.

**Results:**

A total of 33 pertinent aspects of health were identified. Fifteen stakeholders from the qualitative and Delphi studies participated in the prioritisation meeting: 3 young people, 5 parent carers, and 7 health professionals. Aspects of health that emerged as more important for families and targets for health professionals were: communication, emotional wellbeing, pain, sleep, mobility, self-care, independence, mental health, community and social life, behaviour, toileting and safety. Whilst available PROMs measure many aspects of health in the ICF, no single PROM captures all the key domains prioritised as for children and young people with neurodisability. The paucity of scales for assessing communication was notable.

**Conclusions:**

We propose a core suite of key outcome domains for children with neurodisability that could be used in evaluative research, audit and as health service performance indicators. Future work could appraise domain-specific PROMs for these aspects of health; a single measure assessing the key aspects of health that could be applied across paediatric neurodisability remains to be developed.

Health services are increasingly focused on measuring and monitoring outcomes, particularly those that reflect patients’ priorities [1, 2]. Such outcomes include routine data indicators, clinical assessments, and patient reported outcome measures (PROMs in UK; PROs in USA). PROMs are also advocated for use in clinical trials [3, 4], and to incorporate individualised feedback from PROMs into routine clinical consultations [5]. To be meaningful, outcomes measured should be valued by patients and carers, be consistent with what health professionals seek to achieve, and be robust in terms of measurement properties. In the UK, but probably salient elsewhere, there is recognition that families and health professionals do not always share a vision for what services are seeking to achieve, and efforts should establish a common focus [6]. Recent policies underline the growing prominence of outcomes as a mechanism for improving services for children and young people [7, 8]. There appears a strong case for agreeing key health outcomes for pragmatic groupings of children and young people, and identifying appropriate and robust PROMs to measure these areas.

Addressing these objectives is the focus of the Core Outcome Measures in Effectiveness Trials (COMET) Initiative, seeking to overcome the problems of synthesising research when different outcomes and measures have been used [9]. The COMET process engages key stakeholders to seek consensus on ‘what’ domains to measure and ‘how’ to measure them. A World Health Organization initiative to establish International Classification of Functioning, Disability and Health (ICF) ‘core sets’ for specific conditions pursues much the same goals [10].

Neurodisability is an umbrella term for conditions associated with impairment of the nervous system and includes cerebral palsy, autism, epilepsy [11]. Individually, many conditions that result in neurodisability are rare, whereas when grouped together they are common; many of the conditions give rise to similar health issues. Although it is reasonable to develop core outcome sets for more common neurodisability conditions, such as have been proposed recently for cerebral palsy [12], it would also be efficient to devise core outcome sets that could be used across paediatric neurodisability. This would avoid expending limited resources to develop multiple core outcome sets for the vast number of different neurodisability conditions and syndromes.

This paper describes the culmination of research that sought a shared vision regarding a core suite of health outcomes for children and young people with neurodisability, beyond mortality and morbidity. Preliminary work comprised three work streams: (i) qualitative research with service users to identify valued aspects of health from the perspectives of young people and parents [13]; (ii) a Delphi survey with health professionals to identify the aspects of health they target commonly [14]; (iii) a systematic review of existing multidimensional PROMs that could be used across paediatric neurodisability conditions [15]. The ICF Version for Children and Youth (ICF-CY) [16] provided a common language to analyse and compare aspects of health across the different work streams (Fig. 1). The study was conceived to inform health services through informing the development of the NHS Outcomes Framework [1], and was subsequently catalogued by the COMET database [17].

**Fig. 1:**
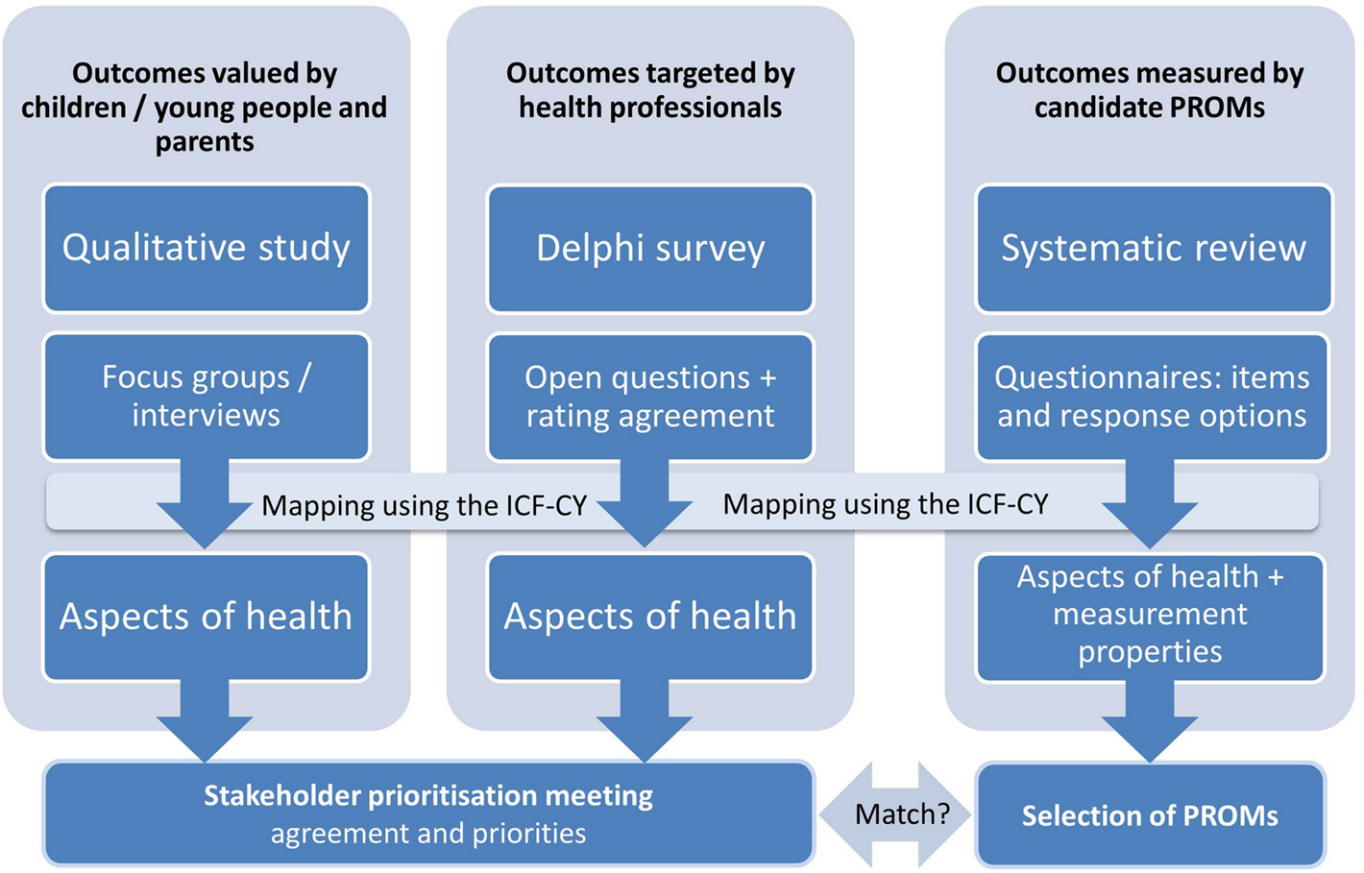
Illustration of study design

In the qualitative study 54 children with neurodisability, and 53 parents participated in either focus groups or interviews [13]. The outcomes discussed by families were: communication, mobility, pain, self-care, temperament, interpersonal relationships and interactions, community and social life, emotional wellbeing, and gaining independence/future aspirations. Parents also discussed sleep, behaviour and safety.

In the Delphi survey the key domains agreed as relevant by over 200 health professionals were: mental health, confidence/emotional stability, anxiety/attention, sleep, pain, toileting, movement and manual ability, acquiring skills, communication, mobility, self-care, recreation and leisure [14]. Although professionals highlighted participation outcomes, aspects of social functioning were perceived generally as less of a responsibility for the NHS.

The systematic review identified 41 multidimensional PROMs that assess various aspects of health in the ICF-CY [15]. Domains covered more commonly are: mental functions, interpersonal interactions and relationships, major life areas, and community, social and civic life [15].

The final stages involved a meeting with a diverse group of stakeholders to seek agreement on core set of ‘shared priorities’ regarding the aspects of health identified. Then to appraise whether any existing PROM could measure the key outcomes prioritised.

## Methods

### Public involvement

The research had a strong ethos of public engagement throughout [18]. Five parents volunteered to be involved; all were parent carers of children with neurodisability (including cerebral palsy, acquired brain injury and epilepsy). Parents worked alongside members of the research team at various stages of the research in co-investigator meetings, parent advisory meetings and in the dissemination of findings. The group did not receive formal training for their involvement but were supported by members of the team; their contribution and traveling expenses were reimbursed.

### Stakeholder prioritisation

We convened a representative group of stakeholders who had taken part in earlier stages of the research. Participants included a range of health professions, young people and parent carers. Health professionals were selected from those who had participated in all four rounds of the online Delphi survey, and a further selection was made to include a range of the different professions by drawing names out of a hat.

The stakeholders were in essence a ‘nominal group’ [19], and the main activity was a Q-sorting prioritisation task [20, 21]. We did not set out to apply the statistical methodology of Q-sorting. Rather, we used the Q-sorting task to observe the processes and discussions that the participants went through when prioritising health outcomes in order to gain insight in to their various attitudes and beliefs.

In advance of the meeting, a list of the health outcomes was compiled from those issues identified (i) from the qualitative research with children and young people and parents; (ii) through the online survey with health professionals; and (iii) from the content of eligible PROM questionnaires identified in the systematic review (Table 1). Duplicate aspects of health were removed. The final list of 33 ‘aspects of health’ or ‘health outcomes’ were represented on laminated cards, with an illustration inspired by the signs from the Talking Mat system used with young people with communication difficulties in the qualitative research. There were some aspects of health that overlapped conceptually, but a decision was made to present these items individually and allow the participants to decide if they should be collapsed as a grouped concept. Nominal group methods involve structured discussion in which participants articulate and clarify their personal views to other members of the group [19]; in our meeting each aspect of health was presented consecutively by a participant followed by facilitated discussion and negotiation about its importance relative to other aspects of health.

**Table 1:** Aspects of health used in the prioritising exercise and sources

Cards depicting:	Qualitative work with children and parents	PROMs	Delphi survey with health care professionals
Play	x	x	x
Sport	x		x
Independent	x	x	x
Communication	x	x	x
Memory	x		x
Concentration		x	
Emotional wellbeing	x	x	x
Fitness and stamina		x	x
Breathing			x
Learning	x	x	x
Let me decide	x	x	x
Muscle strength			x
Moving my body	x	x	x
Manipulating objects		x	x
Moving about	x	x	x
Pain	x	x	x
Worried	x	x	
Personality/confidence and self esteem	x	x	x
Family	x	x	x
Friends	x	x	x
Hearing and seeing	x	x	x
Self care	x	x	x
Eating nutrition	x	x	x
Self care Hygiene	x	x	x
Safety	x	x	x
Sexual health		x	x
Sleep	x	x	x
Social life/go out	x	x	x
Continence	x	x	x
Education	x	x	
Drool, swallowing, constipation	x		x
Body structures	x		x
Control behaviour	x		x
Change body position			x

The card depicting “safety” was not printed and therefore not used in the exercise

**Fig. 2:**
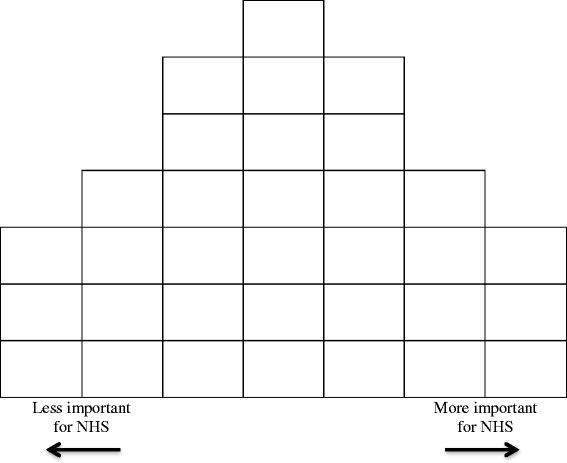
Q-sorting grid used to prioritise aspects of health

To prioritise the aspects of health we split the participants into two groups, each with a mix of clinician and family representatives. They were directed to work collectively to order the 33 cards on a ‘forced choice frequency distribution’ grid (Fig. 2). The column boxes (left to right, x-axis) indicated priority ranking from ‘less important’ to ‘more important’ for the NHS; boxes within columns (y-axis) were assumed to be of equivalent importance, and no item or box indicated unimportance. The final positions of the cards on the grids were used to indicate shared higher (right side columns) and lesser priorities (left side columns). The groups worked through the exercise for 90 min at separate ends of the same large room, and then broke for lunch. The groups reconvened after lunch for 15 min to review their decisions about relative placements of cards on the grids. Finally, the groups came together to compare which aspects of health had been prioritised, and make any final comments.

### Synthesis of findings

We compared the aspects of health identified through the qualitative research and Delphi survey, and those prioritised in the stakeholder meeting. To bring together a core suite of health outcomes relevant to the NHS we identified (i) higher priority aspects of health from the stakeholder meeting, (ii) aspects of health more valued by participants in the qualitative study, (iii) aspects of health targeted by professionals in the Delphi study. Finally, we examined whether these aspects of health can be assessed for children with neurodisability using current multidimensional PROMs.

The NRES Committee North East - County Durham & Tees Valley approved the procedures for this study (reference 11/NE/0364).

## Results

### Stakeholder group

Participants in the stakeholder prioritisation meeting were three young people with neurodisability (one with autism, two with neuromuscular conditions who were wheelchair users), five parent carers of children with various complex conditions including one or more of cerebral palsy, autism, epilepsy, learning difficulties, and seven health professionals: two paediatricians, physiotherapist, occupational therapist, nurse, paediatric surgeon, and a child and adolescent psychiatrist.

### Group dynamics and discussion

**Fig. 3:**
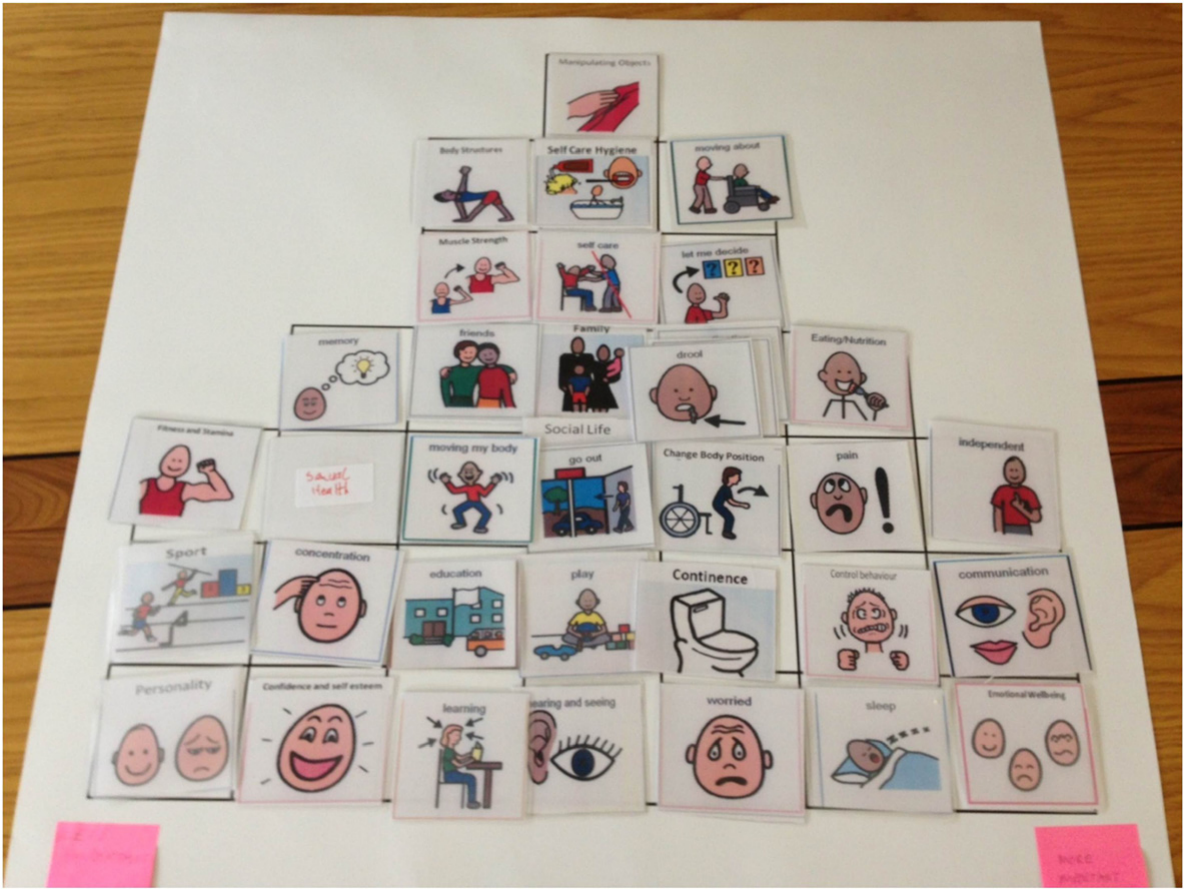
Completed prioritisation grid from one group

The groups engaged well and completed the task (Fig. 3), but found it challenging. There was debate about several aspects of health, for example whether or not they were eligible as being ‘morbidities’ (e.g. seizures) or perceived as self-reportable.

Various issues arose in the process of prioritising concepts as an important focus for the NHS. Participants recognised some concepts as being more readily influenced by health services. They distinguished other concepts as broader ‘life outcomes’ that would be influenced by many factors. Physical outcomes were perceived as more amenable for health services to influence, including ‘pain’, ‘communication’, ‘continence’ and ‘moving my body’. Broader life outcomes that might be harder for health services to influence included ‘emotional well-being’, ‘making decisions’, ‘friends’, ‘family’, ‘social life’ and ‘play’.

### Prioritisation task

There was variation in the ordering of the aspects of health between groups, but communication and emotional wellbeing were both ranked highest. Also ranked highly in both groups were pain, sleep, moving about, independence and worry.

### Synthesis of findings

Aspects of health selected by stakeholders as shared priorities were communication, emotional wellbeing, pain, sleep, mobility, self-care, independence, mental health, and social activities. In addition, the findings of the qualitative and Delphi studies both support behaviour, toileting, and safety as outcomes that are important for parent carers and also targeted by health professionals. Thus we propose these as a suite of important health domains that should be assessed for children with neurodisability, defined with illustrative examples to aid interpretation (Table 2).

**Table 2:** Proposed core suite of aspects of health to be assessed using PROMs for children and young people with neurodisability

ICF domain	Example areas
Communication	Receiving and producing messages, including conversations and using technology
Emotional Wellbeing	Feelings of contentment, enjoyment, happiness
Pain	General or localised discomfort
Mobility	Moving around and changing locations
Self-care	Diet, exercise, washing, dressing
Independence	Expressing preferences, making choices
Community and social life	Recreation, sports and leisure
Mental Health	Anxiety, mood
Sleep	Onset, duration and quality of sleep
Behaviour	Managing expression of emotions, maintaining social interactions
Safety	Managing risks that lead to injury or harm
Toileting	Managing continence, constipation

We examined whether existing multidimensional generic PROMs identified in our systematic review [15] measure these health outcomes. Whilst available instruments measure many aspects of health across the ICF [15], no single PROM captures all the domains prioritised as key health outcomes for children and young people with neurodisability. The paucity of scales for assessing communication was notable. In addition, measures of mobility tend to focus on walking and running rather than the ability to move around independently, which might include using assistive technology to achieve participation in this domain.

## Discussion

Agreement emerged regarding a core set of health outcomes appropriate to assess for children and young people with neurodisability: communication, emotional wellbeing, pain, sleep, mobility, self-care, independence, social activities, mental health, behaviour, toileting, and safety. In defining these concepts we drew on the WHO terminology; each aspect of health is defined in the ICF except emotional wellbeing and independence. No existing multidimensional PROM that can be used across neurodisability assesses all the key constructs. The paucity of scales to assess communication in multidimensional PROMs is a notable omission for paediatric neurodisability. This was an aspect of health that all stakeholders appeared to value highly, as it enables people to express preferences and make choices. The domain-specific ‘Focus on the Outcomes of Communication Under Six’ is a recently developed parent-reported measure of communication participation that informs how this domain can be measured [22]. It is interesting to note that the ICF core set proposed for cerebral palsy did not include communication [12], which was commented on at the time [23]. Given the high priority given to communication in our study this may merit reconsideration.

Our findings provide an incremental step towards a shared vision between families and clinicians of health outcomes for children with neurodisability. Our stakeholder prioritisation meeting showed that disabled young people, parents and professionals can work collaboratively, given appropriate motivation, environment and support. This approach would seem a valuable and important exercise that could be replicated.

Children and young people with neurodisability depend on services across health care, from primary and community care to specialist centres. The findings are important for a wide audience of clinicians, managers and commissioners, and especially those responsible for ensuring health services meet their goals efficiently.

Increasingly, integrated education, health and social care services are advocated as likely to be more efficient and family-centred [24], which creates complexity. Many health professionals in our research felt that there were limitations to the extent to which health services could be responsible for aspects of social participation. This view is consistent with Wilson and Cleary’s model linking clinical variables to measures of health related quality of life [25]. This also confirms our hypothesis based on results from the Delphi survey, where we stated that professionals might perceive themselves ‘accountable’ more for medical issues rather than social participation and well-being [14]. From the perspectives of families who receive support from a range of services, partitioning NHS outcomes may lack credibility; especially as they articulated how aspects of health were perceived as inter-related [13].

Our methods could be modified when seeking agreement on core outcome sets for other conditions, as advocated by the COMET Initiative [17], and when seeking PROMs to measure those outcome domains. The cognitive task for our prioritisation was challenging due to the large number of concepts that had to be understood, recalled and ranked. It has long been recognised that people find it difficult to discern more than a handful of categories [26]. Therefore it would be preferable to conduct a preliminary activity to reduce the number of issues for the final sorting task. Our methodology can be contrasted with procedures recommended to devise ICF core sets for particular conditions that involve qualitative research, Delphi survey and a consensus meeting [10]. The consensus meeting in the ICF process is a residential event with time allocated to training, and iterative voting to rank aspects of health. However, while the experts invited to these meetings come from diverse professions, and often parts of the world, the voices of patients and carers appear less represented; although we note one parent participated in the meeting focusing on cerebral palsy. Sticking strictly to ICF health domains also runs the risk of omitting outcomes such as emotional wellbeing that are conceptually not classified in the ICF.

Our methodology was successful and we engaged a diverse group of stakeholders in the qualitative study, Delphi survey and prioritisation meeting. However, a criticism that can be levelled at our study is that those who took part in the earlier stages were self-selecting, and the prioritisation meeting was a small sample of stakeholders at a single event. One could also debate the right proportions of each stakeholder group. Hence the extent to which our findings can be generalised is debateable, and we welcome further discussion with the broader stakeholder community. The COMET group have identified a number of ‘issues to consider’ when seeking consensus in this context, but do not provide specific guidance [27]. Hence it is difficult to know the ‘correct proportion’ of stakeholders to consult, or how many meetings are required. Therefore testing and reporting transparently our methods and experiences in this study adds to the methodological research in this area.

Another potential limitation is that our approach was non-categorical across neurodisability; therefore our proposed list of key outcome domains may omit aspects of health specifically important for particular conditions. Nevertheless, our findings accord with other research examining key outcomes for children and young people with complex healthcare needs, autism, cerebral palsy, and those who do not use speech for communication [28–31]. Further research could explore whether our proposed core outcomes are replicated in other studies. Such work might explore whether priorities vary for subgroups, for example acquired versus developmental neurodisability and in different age groups.

In our appraisal of generic PROMs, like others, we found poor evidence for reliability of proxy-reports compared to children in many aspects of health [32]. There will always be children and young people who do not have the developmental cognitive capacity to self-report, and it is usually parents and carers who seek health care for their children. Therefore, parent-proxy report is appropriate and may provide important insights. For each domain we need to consider to what extent a proxy (primary carer) is able to report on that construct; in our earlier study parents suggested they had little idea about ‘how their child feels’ when looking at example items in existing PROMs. [13] Parent carers also raised different issues to children/young people that they think are important to measure, such as toileting, behaviour and safety. The potential for a primary carer measure based on the domains of more importance to parents in ways that they feel they can respond accurately merits consideration.

One way forward could be to review existing ‘domain-specific’ PROMs, i.e. questionnaires that seek to specifically measure the core outcomes identified in our study. It is evident that potential respondents should have a greater role in the development process to enhance the validity and acceptability of any PROM. Rasch analysis can also be used to test for invariance of how items perform across age groups, sex and between different diagnoses; the latter being pertinent with the range of neurodisability conditions. One application for PROMs is to measure outcomes over time. Ensuring that scales are robust to measure across different age-groups is important to enable outcomes to be monitored longitudinally. One area requiring further thought is how use PROMs to measure and monitor outcomes for young people as they transition into adulthood, and whether existing adult PROMs assess appropriate aspects of health. A fuller discussion of some of the issues can be found in the full report for this project [33].

## Conclusions

There is much work to be done to realise the potential benefits of using PROMs for children generally [34], and especially with children with neurodisability conditions.

There appears a strong case for further research to develop PROMs for children and young people with neurodisability, especially if their application as robust routine indicators and outcome measures for evaluative research is to be realised. As a next step, it would seem appropriate to appraise existing ‘domain-specific’ PROMs for use with children and young people with neurodisability; that is, those questionnaires that specifically measure the key outcome domains. It is evident from our previous work, and other associated research, that service users should have a greater role in the development of any PROM. The International Society for Pharmacoeconomics and Outcomes Research has recommended good practices for developing PROMs for children and young people which are a valuable resource to guide research [35]; most important, perhaps, is that stakeholders be included as partners from the outset.
